# Bedtime routines in Greek families: characteristics, barriers, and facilitators for establishing and maintaining optimal routines

**DOI:** 10.3389/frsle.2024.1339561

**Published:** 2024-02-08

**Authors:** Marina Papadopoulou, Maria Sandalidou, Ioannis Kamarligkos, Nikolaos Kitsakis, Maria-Aggeliki Milonaki, Frideriki Zografou, George Kitsaras

**Affiliations:** ^1^Department of Psychology, School of Philosophy, Aristotle University of Thessaloniki, Thessaloniki, Greece; ^2^Division of Dentistry, Faculty of Biology, Medicine and Health, The University of Manchester, Manchester, United Kingdom

**Keywords:** bedtime, sleep, routines, child, parents, development, Theoretical Domains Framework

## Abstract

**Background:**

Bedtime routines are amongst the most common, recurrent family activities with close associations for child health, development and wellbeing especially sleep. Despite the importance of bedtime routines, no previous study has examined them within the context of a Greek family.

**Method:**

A mixed methods, stepped approach was used in this study. A cross-sectional study examined the prevalence and characteristics of bedtime routines (PRE) in families with young children and explored associations with parental mood (POMS) and child's sleep (CSHQ). A qualitative study using the Theoretical Domains Framework (TDF) examined barriers and facilitators for achieving optimal routines.

**Results:**

Total of 54 parents with a mean age of 35.9 (SD = 5.95) completed the cross-sectional study while 20 parents participated in the interviews. There were strong positive correlations between total scores on the POMS and total scores on the CSHQ *r* = 0.482, *p* < 0.001 and strong negative correlation between total scores on the POMS and total scores on the PRE, *r* = −0.308, *p* = 0.023. In terms of barriers and facilitators, social desirability, social comparison, environmental factors and resistance from children were amongst the most common barriers to establishing a good bedtime routine.

**Conclusion:**

Bedtime routines are highly prevalent in Greek families with the quality of those routines varying between households. Addressing common barriers in achieving better bedtime routines could help families benefit in the short and long-term.

## Introduction

Family routines play a significant role in family life. Past research has shown the benefits of repetitive, predictable proximal processes for children's development (Spagnola and Fiese, 2007). Repetitive activities on a daily basis provide safety, promote learning and help establish good and long-lasting habits (High et al., 1998; Spagnola and Fiese, 2007; Kitsaras et al., 2018). From a wide range of family routines, bedtime routines are an essential part of family life. Bedtime routines incorporate a range of behaviors from interactive to non-interactive activities (Mindell and Williamson, 2018). An optimal bedtime routine incorporates activities such as oral hygiene practices before bed, avoiding sugary and other snacks/foods before bed, limiting the use of electronic devices (such as watching TV, using laptops/computers and gaming consoles), reading or sharing a story before bed, and calming parent-child play (Davies and Bridgman, 2011; Mindell and Williamson, 2018; Kitsaras et al., 2021a). Consistency, within reason, is another vital component of optimal bedtime routines with routines adapting to children's developmental stages (Levine, 2001; Fiese, 2002; Kitsaras et al., 2018, 2021a; Mindell and Williamson, 2018). Not every family will incorporate all these activities as part of their bedtime routine and routines will have a degree of flexibility to accommodate personal and societal differences depending on where a family lives, who else lives in the same household etc.

As with other facets of family life, bedtime routines are only one factor potentially influencing family dynamics as well as parent and child-based outcomes. Multiple external factors (i.e., socio-economic factors, demographics, where one lives, access to services, political changes and many more) can all affect individuals and families alike (Teachman, 2000; Conger et al., 2010). Routines are not standalone behaviors but rather a reflection of multiple internal and external processes some not in the direct control of the parents. However, in many countries around the world, most families will have a routine in place around bedtime with some cultural variations on what each family routine involves (Mindell and Williamson, 2018). As Mindell and Williamson (2018) showed in their work, bedtime routines appear quite stable across different countries especially countries that will fall under the definition of “Western Developed Economies” (such as the UK, US, Canada, Australia and EU member states) with main differences found on the time children go to bed, where they sleep, how many people sleep in the same room and what time children wake up. While reading before bed is common in many countries, storytelling, talking with parents/grandparents and even siblings while in bed are all common activities children undertake during bedtime (Mindell and Williamson, 2018). Equally, interactive and/or soothing activities are present in different countries but may vary from bath before bed to praying and/or rocking, massaging etc. (Mindell and Williamson, 2018).

Even though bedtime routines are important and relevant for all ages and types of families, first-time parents with young children are of particular interest. This group has no previous experience of establishing and following a bedtime routine with children. Therefore, if they manage to establish optimal routines with their first child, then any future children can also benefit from optimal routines (Kitsaras et al., 2021a). The principle of “learning some first, learning it right” is crucial for bedtime routines as with other parental and family activities (Olds et al., 2013). Families expecting their first child and first-time parents in general are uniquely placed to receive beneficial advice and support when they need it rather than trying to alter established yet suboptimal behaviors and practices later on (Olds, 2006).

Some form of routine around bedtime can have beneficial impact on children's wellbeing, health and development (Hale et al., 2011; Mindell and Williamson, 2018). Previous research has shown that good bedtime routines provide benefits for children in terms of better health (i.e., quality of sleep, dental health) (Mindell et al., 2009; Kitsaras et al., 2021c), cognitive functions including better school performance (High et al., 1998; Kitsaras et al., 2018) and emotional aspects of psychosocial development (Mindell and Williamson, 2018). Alongside children's wellbeing, there is evidence to support that optimal bedtime routines reduce parental stress, decrease parental mood disturbances, increase confidence, and promote overall family wellbeing (Evans and Rodger, 2008; Mindell and Williamson, 2018). More stable, less argumentative and disrupted routines can lead to better overall family functioning.

Sleep is of particular interest when it comes to bedtime routines with the majority of existing work focusing on the relationship between bedtime routines and sleep (Mindell and Williamson, 2018). Sleep is an important facet of healthy life with disrupted sleep affecting childhood development and wellbeing from poor developmental outcomes such as socio-emotional development (Hysing et al., 2016) and neurocognitive health (Smithson et al., 2018; Mason et al., 2021). Child sleep problems including difficulty falling asleep, night waking and shortened sleep duration are highly prevalent with around 20% of young children experiencing issues around sleep (Williamson et al., 2019). One of the key recommendations in addressing sleep-related issues in children is establishing and maintaining a stable bedtime routine (Mindell and Williamson, 2018).

Despite being a much-discussed topic for a lot of parents, there is a lack of studies focusing on characteristics and prevalence of bedtime routines among different population groups. Research that examines differences between countries, cultures, socioeconomic status and family types, is much needed. For example, there is little to none research on bedtime routines from the perspective of Greek families. At present, no specific recommendations exist regarding bedtime routines for families in Greece. Published research in Greece focus primarily on sleeping patterns from a medical perspective (Lazaratou et al., 2005; Paraskakis et al., 2008; Nena et al., 2020; Kanellopoulou et al., 2021). Nevertheless, initial yet limited evidence highlight a different pattern regarding bedtimes in Greek families for example, the time children go to bed in Greece compared to other counties such as the UK (Evans and Rodger, 2008; Mindell et al., 2009; Hysing et al., 2016; Kitsaras et al., 2021c).

### Objectives and aims

The main aims of this study are: (a) to explore the prevalence of bedtime routines in families with young children living in Greece and (b) to understand key barriers and facilitators for establishing optimal bedtime routines using a standardized framework that allows for intervention considerations in the future. The main objective of this study is to contribute to the scientific knowledge about the bedtime routines in general and to reduce the knowledge gap for Greek families in particular.

## Materials and methods

A stepped study design was utilized in this study. Firstly, a cross-sectional study examined the prevalence and characteristics of bedtime routines in families with young children. Secondly, a follow-up qualitative study examined barriers and facilitators regarding the implementation of bedtime routines in the same families.

### Study 1

#### Participants and recruitment

For the purpose of this study a total of 54 parents with children aged between 1 to 5 years of age were recruited. Inclusion criteria were: (1) having a child between the ages of 1 and 5 years old and (2) having only one child. A convenient sampling strategy was deployed for the study. Participants were approached, online or in person, first through family and friend networks (through word of mouth approach) and in a small number of cases through professional relationships/settings (e.g., places of work, colleagues etc.). Later on, social media posts (e.g., Facebook) were also utilized for a wider participant reach. All participants were provided with an overview of the study (in the case of social media, a summary of the study was shared online to attract interest) and offered sufficient time to consider if they want to take part. All participants provided informed, written consent if they decided to be included in the study. Participation in both studies was voluntary and no compensation was provided to participants. All elements of the study were approved by the Ethics Committee of the Department of Psychology at the Aristotle University of Thessaloniki (Ref.: 059/06-05-2022).

#### Data collection

Data collection took place from April to June of 2022. Online questionnaires were completed by one parent representing one family. Data was collected electronically through the following questionnaires: (1) Abbreviated Profile of Mood States (POMS) Questionnaire (Shacham, 1983) as translated and used in previous studies with a Greek sample (Papastergiou et al., 2018), (2) Children's Sleep Habits Questionnaire (abbreviated) (CSHQ) (NICHD SECCYD-Wisconsin, 2014) with items copied from a validated translation of the long-form CSHQ with a Greek sample (Mavroudi et al., 2018), and (3) Bedtime Routines Questionnaire (PRE) (Henderson and Jordan, 2010). The PRE questionnaire was translated in Greek from the study team as there has been no previous translations of the scale.

POMS is a widely used, validated and reliable instrument for capturing mood disturbances in general population (Searight and Montone, 2020). POMS contains 40 statements that participants score using a 5-point Likert scale (not at all, a little, moderately, quite a lot and extremely) producing seven subscales: parental tension, anger, fatigue, depression, self-esteem, vigor and confusion. Total negative scores (tension, anger, fatigue, confusion and depression) are added together and then the positive scores are subtracted subtracting the total positive scores (self-esteem and vigor) to produce the total mood disturbance score where higher scores indicate higher parental mood disturbance (i.e., greater negative mood). CSHQ is one of the most commonly used peadiatric sleep validated questionnaires (Lazaratou et al., 2005). It contains 22 questions answered on a 5-point Likert scale (always, usually, sometimes, rarely, and never) and produces 4 subscales relating to children's sleeping habits: bedtime routine activities, sleep-related behaviors, night waking and morning wake up behaviors. Finally, the bedtime routines original questionnaires contains 31 questions on a 5-point Likert scale (almost never, occasionally, half the time, often and nearly always) spread across four target areas: weekdays, weekends, how upset the child gets if he or she does not perform some activities and a list of 15 bedtime-related activities. However for this study, an adaptive version was used with 10 overarching areas of interest better reflecting the activities taking place at bedtime. Despite merging questions, the 5-point Likert scale was still followed for participants' responses. All participants completed a brief demographics form at the start of the study.

#### Data analysis

Questionnaire responses were scored using the relevant scoring key. For POMS, Total Mood Disturbance (TMD) score is calculated by summing the totals for the negative subscales (tension, depression, fatigue, confusion, anger) and then subtracting the totals for the positive subscales (vigor and esteem-related affect). For the abbreviated version of CSHQ, scores were calculated for: bedtime behaviors, sleep behavior, night and morning waking as well as a total score. Finally, for the PRE, scores were calculated for weekday and weekend consistency of routines as well as for bedtime routines activities.

Descriptive statistics were analyzed to provide an overview of key data points. Descriptive statistics were used to provide an overview of our sample characteristics and mean results across our key data points. Histograms were also calculated for some core outcomes. Pearson correlations were used to examine associations between parenting mood, bedtime routines and children's sleep habits. All analyses were conducted on SPSS v.27 (IBM 2022).

### Study 2

#### Participants and recruitment

A total of 20 parents who completed study 1 were recruited for this study. A snowball method was followed in order to reach the desirable goal of 20 interviews. Data saturation was monitored to ensure that all questions were sufficiently covered.

#### Data collection

Interviews were conducted online or on the phone. In each interview, a detailed semi-structured interview schedule based on the 14 domains of the Theoretical Domains Framework (TDF) (Cane et al., 2012) was used. Each TDF domain was explored with a combination of different questions. The average duration was 40 min.

#### Data analysis

Interviews were transcribed verbatim using a transcription service. A deductive analysis was carried out according to the Theoretical Domain Framework (Cane et al., 2012). Two independent coders used a deductive approach to map each statement to one of the TDF domains. Barriers and facilitators were identified based on participants' responses. Overarching themes were also identified and summarized (while frequency counts were used to determine the most commonly endorsed domains and specific component constructs).

## Results

### Study 1

#### Sample characteristics

Total of 54 parents with a mean age of 35.9 years (SD = 5.95) completed a series of online questionnaires. Forty-four female and 10 male participants took part in the study. Out of the 54 participants, seven were unemployed, five in part-time employment, 39 in full-time employment with one participant neither employed nor unemployed. Most participants (48 out of 54) had some University-level education at either undergraduate or postgraduate level with 1 participant holding a doctorate. Additionally, 4 participants had high school education and 1 participant reported “other” in their education history. Finally, out of 54 children, there was an almost equal split between boys (*N* = 28) and girls (*N* = 26) while the average age of children was 2.7 years (SD = 1.25).

#### Prevalence of bedtime routines

All participants in the study had some form of bedtime routine in place. In terms of time children went to sleep, on average children went to sleep around 9.30pm (M = 9.50, SD = 0.82) on weekdays and around 9.45 pm (*M* = 9.78, SD = 0.74) on weekend. The earliest children went to bed was 8.00 pm and 11.30 pm was the latest reported. There was no statistically significant difference in the time children went to sleep between weekdays and weekends contrary to previous studies where a weekend effect was identified. Also, there was no statistically significant differences in the time children went to bed based on their age. In terms of the characteristics of their bedtime routines, mean scores on PRE was 56.68 (SD = 5.69) with high scores indicating more optimal bedtime routines. The adapted version of PRE used in the study could produce a maximum score of 80, the vast majority of participants, apart from one, scored over 40 in the PRE with 19 participants scoring 60 or over.

In terms of different bedtime routine activities, these were scored across seven areas: reading before bed, playing with toys, interacting with children before bed, watching TV before bed, playing with electronic devices before bed, eating/drinking before bed, brushing teeth and looking after teeth before bed and finally, having a bath or a shower before bed (personal hygiene). Table 1 summarizes the frequency of each of the seven core bedtime routine activities. In terms of reading before bed, 44.4% (*N* = 24) of parents read to their children nearly every night. Almost half of parents, 48.2% (*N* = 26) allowed their children to play with their toys before bed while a large majority of 73.3% (*N* = 45) interacted with their children nearly every night. Some parents did not allow their children to watch TV before bed (46.3%, *N* = 25) nor to eat or drink something before bed (48.1%, *N* = 26) while the majority of parents did not allow their children access to electronic devices, apart from TV, before bed (81.5%, *N* = 44). Finally, most parents brushed their children's teeth before bed nearly every night (51.1%, *N* = 33) and looked after their children's personal hygiene through a bath or a shower before bed (63%, *N* = 34).

**Table 1:** Summary of bedtime routine activities' frequency in percentages.

	**Reading**	**Playing toys**	**Interaction**	**TV**	**Electronics**	**Food/drink**	**Oral health**	**Bath**
Almost never (%)	12	14	1	25	44	26	9	5
Occasionally (%)	9	9	4	7	6	15	8	2
Half of the time (%)	9	5	4	8	3	6	4	13
Often (%)	12	15	39	5	0	3	23	23
Nearly always (%)	12	11	6	9	1	4	10	11

#### Associations between parental mood disturbance, children's sleep and quality of bedtime routines

Pearson correlations were calculated to examine any initial associations between parental mood disturbance (calculated by total mood disturbance scores on the POMS), children's sleep (calculated by total sleep scores on the CSHQ) and quality of bedtime routines (calculated by the total scores on the adapted version of PRE). There were strong positive correlations between total scores on the POMS and total scores on the CSHQ *r* = 0.482, *p* < 0.001. In both questionnaires, higher scores relate to mood disturbance and poor sleep. There was also a strong negative correlation between total scores on the POMS and total scores on the PRE, *r* = −0.308, *p* = 0.023 with worse routines resulting in higher parental mood disturbance. There was no statistically significant association between total scores in children's sleep and quality of bedtime routines however, bedtime routines showed significant negative correlations with one subscale of the CSHQ such as morning waking *r* = −0.332, *p* = 0.014.

### Study 2

#### Sample characteristics

Total of 20 parents with a mean age of 35.4 (SD = 5.79) took part in an interview. There were 19 female participants and 1 male. Nineteen people of the total sample were married and living with their partners, and one was separated. Out of 20 children, there were 14 girls and 6 boys, while the average age of children was 2.95 years (SD = 1.39).

#### TDF-based analysis

A thematic analysis was carried out focusing on the 14 factors of the TDF and broader themes relating to barriers and facilitators to bedtime routines. A deductive approach was used to map each statement to one of the TDF domains or outside of the TDF. Based on the result of the analyses, all TDF domains were sufficiently covered by participants' replies. Each domain is presented below with a selection of participant quotes. Overarching themes were identified as well as key barriers and facilitators for the target behavior (bedtime routines).

##### Knowledge

New parents seem to navigate parenting with the help of the internet and their support systems. They all seem to have some, at least initial, knowledge what to do around bedtime. Parents seem to take a proactive stance where they search online for information on how to achieve better bedtime routines as well as having conversations with family/friends. Sources of information also include books and advice from healthcare professionals. However, the dominant source of knowledge seems to be online resources.

“*…the truth is that I was reading a lot of books and advice from other parents who were my age or older… the internet in general was a conflicting and confusing space…” (Q11)*

“*I learned everything from articles not from literature, as in a specific book. From articles, from online magazines that are about parenting etc....” (Q1)*

However, information coming from family or friends allows parents to compare and contrast their own practices with others' in an attempt to find out what could work best for their family.

“*From the internet, from friends, from other mums we've talked to, and they've managed to get their children to actually sleep for nine and a half hours …” (Q2)*

##### Skills

Parents valued patience, perseverance, and time organization the most. As it is mentioned, parental fatigue can play a detrimental role in maintaining a consistent sleep routine for children. Patience, perseverance, and good organizational skills are therefore of the essence for achieving better routines despite the impact of fatigue. Actions such as drawing up a schedule, making appropriate preparations, and generally organizing personal time all seem to be facilitating factors in achieving stable routines.

“*… I think the greatest skill you have to have is patience” “..to be able to distance yourself from your problems...” “Always organize your time.” (Q11)*

“*Yes it takes good organization [skills] to make a schedule for your child… then your child trusts you, … [they] feel comfortable to enjoy the routine with you.” (Q12)*

##### Social/professional role/identity

Most participants seem to adopt a “*savior”* identity; parents feel that the difficulties they encounter in their daily lives are worth it because of the love they have for their children. That love justifies every sacrifice of time and personal space, and creates a feeling of accomplishment.

“*Sacrificing your own so that the child can have his.” (Q2)*

“*She is my child! I love her, so no matter how much she torments me I will be there.” (Q10)*

Mothers seem to carry a disproportionate responsibility around bedtime routines. This finding in part reflects a wider societal focus on women as the main caretaker for children of all ages, especially younger children. Fathers are involved in family life but their involvement tends to fluctuate more when family duties clash with other responsibilities (namely work). In some cases, fathers tend to primarily be occupied with fun activities rather than a more responsible role. This is not necessarily the case for all families, but these results map on a greater societal level of stereotypes in child rearing practices and the expectations placed on women.

“*...I would say that 99% of the routine is me. That is, every night I need to take care of the bath, dressing up the child and so on. My husband helps with some things when he's here because sometimes he works too...” (Q18)*

##### Beliefs about capabilities

Parents generally seem to have confidence in their own strengths and they seem to believe in their own abilities. Establishing a consistent bedtime routine for their children is something they can do, as long as they keep trying, even on the hardest, most tiring days.

“*Usually.. I'm patching up. I have to be more patient I think.” (Q4)*

“*It's pretty easy…except on hard days when she's tired and grumpy and there's tension coming out. But generally easy.” (Q9)*

##### Optimism

Despite difficulties, parents remain relatively optimistic about the future. This optimism is reflected in their belief that children will eventually grow up and be more autonomous. They envision a good future for their children in terms of routine-schedule adherence. They believe that in the future children will be more easily independent, precisely because of the work they put in now.

“*Uh, talking with him, as he gets older to find more things that he likes.. I'll ask him to see what he wants to add … he can substitute something he likes and it helps him relax at that time…” (Q12)*

“*I think that what will change is maybe that he will be able to sleep alone. I think that will happen soon, because now he won't do that.”(Q16)*

##### Beliefs about consequences

Participants seem to be concerned about the consequences of poor sleep. They express concerns that poor sleep can have detrimental effects on children's development. Despite bedtime routines covering a wide range of activities and with implications beyond sleep, getting a good night's sleep was the primary concern for parents.

“*I believe that sleep is very important, especially night sleep. If sleep is not good, I think the child will be more restless, won't calm down, he won't be able to concentrate… He will not be able to sleep and be calm. And I think that sleeps plays a role in everything else [in life].” (Q6)*

“*…he needs to get some hours of sleep [each night]. Otherwise, you find this [problem] in front of you. That's what I think, without getting into the science of it, I think I'm convinced by that argument. [Sleep] is good for her health.” (Q13)*

##### Intentions, reinforcement, and goals

In general, intentions varied with some parents focusing on short-term considerations and others focusing on long-term. Intentions are essential for family life as they provide some structure and an idea of what to expect at different points in the day. Change is part of family life; therefore, how a family reacts to change will determine the family dynamics. Short-term intentions focused more on creating habits for children and generally strengthening the routine with the more gradual participation of the children. Long-term intentions concerned the child's self-determination and discovery of its own interest.

“*Look, when the kids grow up, they definitely have their own habits so to speak, based on their character. What they might like best or something they want to do before they go to sleep… So, if you see something that the child needs more, that can be added to the routine in the future… I will definitely add it, and if it's for the good of the child.” (Q7)*

Bedtime routines create positive reinforcements for parents as well as children. Parents' positive experiences can lead to a greater willingness to repeat the target behavior over a period of time and to persist in the face of resistance and difficulties.

“*..well, it's too sweet a routine to let go of. There's a tenderness to it all because we're lying together… it's all familiar. For me, that makes me feel warm and cosy too. It goes without saying that if I happen to do two nights in a row where I can't participate in it, it bothers me, I miss it, and I want it. So, I'm sure that's my main motivation.” (Q13)*

Through the awareness of positive outcomes and reinforcement, parents aim to achieve good bedtime routines for their children and the family as whole. Bedtime is also seen as an important time for parents and children to interact creating stronger bonds and attachment for later life. Finally, parents can see the immediate and tangible benefits of having a routine in place as they can then gain some time for themselves at the end of a busy day. Therefore, maintaining this process becomes an incentive for parents. If all goes well then parents have quality time for themselves.

“*We always want to have a good time with her, we basically want to see her sleeping with a smile on her face. So, we always want to laugh, dance, tease her before she goes to sleep. We want her day to always end beautifully, for the child to sleep beautifully so that she can wake up better.” (Q11)*

##### Emotions

Emotional reactions to bedtime routines span two opposing feelings. On one hand, parents feel stressed and frustrated as bedtime can be a challenging time for them. These feelings are further reinforced by the time constraints, tiredness (physical and cognitive), lack of help from others like their partners, or ‘bad' habits that create resistance. On the other hand, if bedtime routines conclude without issues, parents feel a range of positive emotions such as gratitude, security, peace, calmness and contentment.

“*I am relieved by the stability. For me, the existence of this routine creates a sense of security for me as a mother and I see it in the child that it works.”(Q15)*

“*I personally am stressed by bedtime… I am more comfortable on the days I do not work than on the days I know I am working.” (Q11)*

##### Memory, attention, and decision process

For most parents, and in some cases even for children, their bedtime routine is an automatic process that they do not need to actively recall, as they have already repeated enough times to know the necessary steps. So, the routine is described more as an established habit. The only deviations that can occur and derail this automatic process are in exceptional circumstances for example, around holidays or social events that disturb their family routine.

“*Yes, but after a while, it goes experiential, meaning you remember it, it goes on its own.. you do it automatically.” (Q6)*

“*… he's asking for it too. When he drinks the milk and I tell him to go to sleep, he says alone that we should go to bed and read.” (Q1)*

However, even though routines are more of an automatic process, they are not rigid. As the child grows up, the routine adapts to change and the child takes a more active role in shaping it, expressing his or her own wishes and needs. Although parents see routine mainly as a habit, they recognize the need to respond to ongoing changes and daily challenges as the child develops.

“*I didn't have to remember anything… I was following certain things like a soldier and I was following certain things because the child just didn't have that much of an opinion on what to follow, I was telling her let's go, do this, we do that, we do this, we do that, we do that, it was something that I did automatically and she did it with me. Now as she gets older, she has her own opinion, she doesn't follow me so easily, you have to find ways to get her to follow the routine or you have to adjust it in your head based on what you think will intrigue her to follow.” (Q11)*

##### Environment and resources

Resources, or lack thereof, in homes is recognized as an important issue affecting sleep routines in a variety of ways. In some cases, environmental aspects are mentioned as facilitating factors in establishing and implementing the routine, such as, for example, the existence of sufficient space so that the child to sleep, play etc. Participants also claim that access to resources, such as books/games, which they use in the routine, act as another important facilitator.

“*We start and spend our whole day downstairs [in the house]. We go upstairs when he needs to go toilet, brush his teeth and go to bed. I think it's positive that upstairs in our house is not associated with play .. it is only for sleeping. We put the lights on, some little lights in her room, more relaxing more peaceful things. Of course, as soon as she sees a bed, like all children, she starts jumping up and down.” (Q17)*

“*It has to do with choices somehow. It would be more difficult without books. books play a role.” (Q1)*

Some other parents mention factors of house environment (such as the noise and bustle of the city, or noise due to lack of space and soundproofing in the house, cold etc.) as obstacles to the child's sleep habits and the smooth progression of the routine.

“*but for example we have a road outside the house, and this sometimes causes concern to the baby because cars pass by very fast, with noise and this affects her. She may sleep or want to sleep and be disrupted by the noise.” (Q5)*

“*The only thing that can affect it is the cold in the winter.” (Q6)*

##### Social influences

In terms of social influences, there is a split in the sample with two key themes emerging. Some parents seem to make comparisons between their routines and other families' routines and to consider, to some extent, family and friends' opinions. For some parents, positive comments from others offer reassurance about their routine. Other parents seem to disagree with advice offered from others especially if others' routines are “too strict”.

“*Other mothers I see on the playground have very stable routines. Is it seven o clock? They have left the playground and head home to start their routine. For me, if he plays and has a good time, I'll let him carry on, I'm not so concerned with time.” (Q4)*

Most parents believe that routines are somewhat unique to their family situation and circumstances. Routines respond to different needs and therefore consider that adaptations are necessary to meet their family needs. So, flexibility is a feature of the routine that every family has.

“*Well, no, I don't care what other people think about it because I think that every child is different and the sleep routine has to be adapted, I think it has to be adapted to the family and the needs of each family.” (Q14)*

“*No I don't care what they think, I've been told by some mums, how do you do x, it's stressful, it's binding etc.” (Q12)*

##### Behavioral regulation

To adhere to the bedtime routine, different factors such as family obligations and time need to be considered. Routines also need to take into consideration other factors such as the age and preferences of the children.

“*... I had to set up a lot of things during the day, so I had to plan ahead to achieve our routine that night...sometimes I might need to make sure that no one's coming around late in the evening and wakes up the little one and takes us out of our sleep routine, that is, somehow the whole family's schedule is regulated so that the child, when it's time for him to go to bed he's a little tired, not having a lot of stimuli....” (Q18)*

### Overarching themes

**Figure 1:**
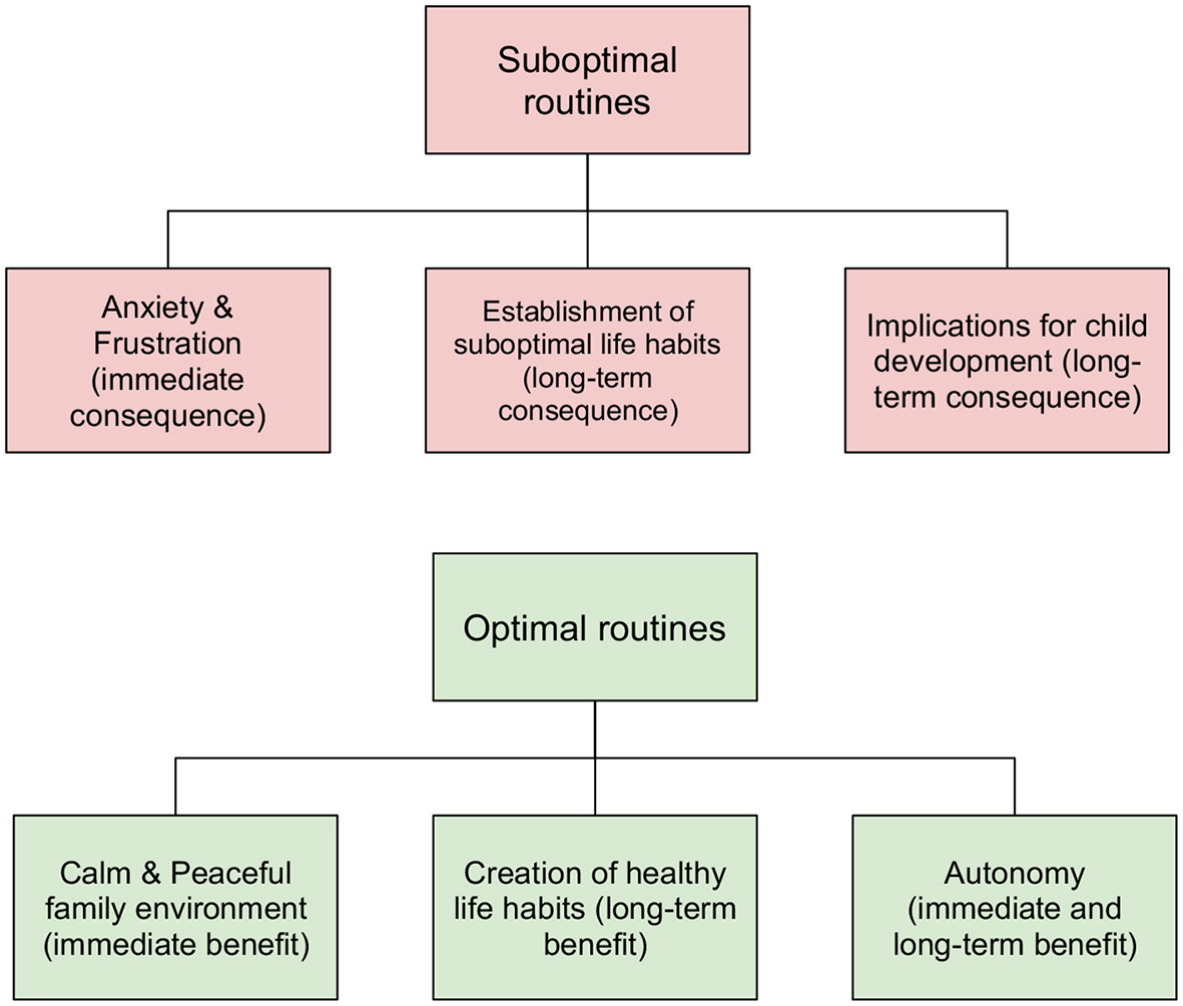
Schematic representation of consequences and benefits associated with suboptimal and optimal bedtime routines respectively.

Reflecting on the different domains, two key overarching themes emerged: (a) negative consequences of suboptimal routines (presented in Figure 1) and (b) positive effects of optimal routines (presented in Figure 1). Both themes combine salient, short-term and tangible outcomes (i.e., anxiety for parents whose children do not have an optimal routine in place v. calm and peaceful family environment for those families with optimal routines in place) as well as long-term, less immediate consequences (i.e., implications for child development associated with suboptimal bedtime routines v. creation of healthy habits for later life for those with optimal routines).

### Barriers and facilitators

As per TDF analysis, key barriers and facilitators for the target behavior (bedtime routines) were identified. These are listed in Table 2.

**Table 2:** Key barriers and facilitators for optimal bedtime routines.

**Barriers**	**Facilitators**
(for some parents) Other parents' sleep routines and wealth of knowledge and information on how to create bedtime routines result in stress and insecurity for parents about their own performance (social comparison and inability to know what to do, how to do it and who to trust)	(for some parents) Availability of knowledge and information sources from the internet
(for some parents) Structural limitations of the house environment interfere with the smooth progression of sleep	(for some parents) Positive environment and availability of resources to facilitate routines (for those households with access to necessary resources)
Children are still young and do not actively participate in the sleep routine resulting in some resistance during bedtime	Involving both parents in sleep routines makes the process more relaxing, easy and enjoyable. However, when such support is lacking, routines can become more difficult.
	Positive reinforcement through engagement with routine (when routines are calm and peaceful). Positive experiences can lead to a greater willingness to repeat the target behavior

## Discussion

In line with previous research outside of Greece (Kitsaras et al., 2018, 2021c; Mindell and Williamson, 2018), bedtime routines are prevalent in first-time parents with young children in Greece too. Routines have a degree of variation with some activities not achieved consistently each night. This is not uncommon as routines fluctuate with family life. Preliminary results from the analysis of this cross-sectional study show how routines, when stable and achieve most of their intended goals, could potentially have a degree of influence over parents' mood as well as children's sleep. Qualitative interviews unearthed some key barriers and facilitators for achieving (or not) optimal bedtime routines each night which could lead to future interventions to improve bedtime routines.

### Prevalence and characteristics of bedtime routines

Similar with countries in Europe, US, Canada and Australia/New Zealand, bedtime routines are a prevalent, recurrent feature of family life for Greek families. Most families had elements of a bedtime routine in place as showcased by the results of the first study. One important difference noted in the Greek sample compared to work in other countries is the time children go to bed. In this study, on average, children went to be around 9.30 pm (*M* = 9.50, SD = 0.82) on weekdays and around 9.45 pm (*M* = 9.78, SD = 0.74) on weekends. The earliest children went to bed was 8.00 pm with 11.30 pm the latest. Comparably, bedtime in Australia, New Zealand and the United Kingdom is around 7.00–8.00 pm for children of a similar age (Mindell and Williamson, 2018). Nevertheless, later bedtimes are common in other countries such as Japan, South Korea, Hong Kong as well as in some Mediterranean countries like Spain (Mindell et al., 2010; Mindell and Williamson, 2018; Cassanello et al., 2021). Cultural differences in the time children go to sleep can be explained in part by the overall societal structure of one's day. For example, later mealtimes, later opening/closing hours for shops and hospitality settings, longer and warmer days in the Summer as well as other parental and child activities that last into the evening bedtime is pushed back to a later time. Research on sleeping patterns of Greek adults and adolescents has highlighted the tendency for later bedtimes across different age groups with one study reporting a bedtime of around 11.00–12.00 pm and even after midnight for some respondents (Paraskakis et al., 2008; Kanellopoulou et al., 2021).

Apart from later bedtimes, there was no statistically significant difference in the time children went to sleep between weekdays and weekends contrary to previous studies where a weekend effect was identified. A weekend effect, whereby bedtime routines start later on non-school days (Friday night and Saturday night) is to be expected as families enjoy more time together with less urgency to have children off to bed at a specific time due to an early/scheduled wake up the next day. This weekend effect has been observed in previous work within the UK (Kitsaras et al., 2021b). In the case of this work, lack of weekend effect could be partially explained by the overall later bedtimes. If children go to bed later every day that will be the case for the weekend too. On average, in this study, children went to bed around 9.30 pm (*M* = 9.50, SD = 0.82) on weekdays and around 9.45 pm (*M* = 9.78, SD = 0.74) on weekend, a very minimal delay.

When examining prevalence of different activities around bedtime, some results are particularly interesting. For example, a very large section of our sample (81.5%) did not allow their children electronic devices the hour before bed. Use of electronic devices before bed can have detrimental effects on sleep (Cain and Gradisar, 2010). Blue light emitted from screens and the rich, stimulating content consumed around bedtime or in bed can lead to decreased melatonin level which in turn delay sleep inhibition and result in disturbed sleeping patterns and overall decreased sleep duration (Staples et al., 2021). Compared to other countries such as Australia where as many as 90% of children and adolescents used an electronic device before bed (Brushe et al., 2022), Greek families in this study seem to be on the exact opposite of the spectrum with only <20% of parents reporting use of electronic devices before bed. Different reasons might be responsible for this contrasting finding such as the age of children in our study compared to other published studies, effects of desirability bias or simply a greater awareness of the effects of screens before bed in this particular sample.

Contrary to use of electronic devices before bed, other bedtime activities were less than preferable. For example, less than half of families in this study reported reading to their children before bed (44.4%) a crucial activity for language and cognitive development (Mindell and Williamson, 2018). Equally, only around half of our sample (51.5%) brushed their children teeth before bed or avoided snacks before bed (48.1%). Oral hygiene practices at bedtime alongside avoidance of food and drink the hour before bed are both associated with better oral health outcomes for children (Selwitz et al., 2007; Davies and Bridgman, 2011; Goodwin et al., 2017; Kitsaras et al., 2021c). Apart from being a crucial aspect of an optimal bedtime routine, oral hygiene practices such as brushing before bed and avoiding snacks can lead to a lower incidence of dental caries (cavities) in children of all ages with young children a group of particular interest regarding establishment of optimal habits for later life. Greek children are not immune to the effects of dental disease with around 17.7% of 5-year-olds experiencing dental caries in Greece (Diamanti et al., 2021). Dental caries is the most common yet preventable non-communicable disease in the world and for children, if left untreated, caries can result in pain, sleepless nights, days off school due to pain, unnecessary, lengthy and costly dental treatment and in some cases dental extractions (Feitosa et al., 2005; Baghlaf et al., 2018; Levine, 2021).

### Bedtime routines, child sleep and parental mood

As this was a cross-sectional study, no direct assumptions can be made regarding the data provided by parents on mood, sleep and bedtime routines. There is some initial data to suggest that parental mood could be affected by the overall quality of bedtime routines. If true, this will be in line with previous work where parental mood disturbance was higher in households where bedtime routines were less stable, with lower level of interactions with parents and children (Mindell and Williamson, 2018; Kitsaras et al., 2022). As bedtime routines occur later in the evening any resistance, tantrums and disturbance can lead to worsen mood for parents and for their children. As parents and children are physically and cognitively more tired in the evening, the effects of bedtime routine-related issues can be exacerbated and result in higher mood disturbance for those involved in the routine. On the other hand, a smooth, timely and incident-free bedtime can lead to better overall mood. Regardless of the effect of bedtime routines, family life is affected by a myriad of external, societal and environmental factors all of which could affect parental mood, children's sleep and ultimately create disturbances in family life (Teachman, 2000; Spagnola and Fiese, 2007; Conger et al., 2010).

One key difference in the results of this study is with regards to the lack of associations between total scores in child sleep and bedtime routines. Literature suggests a strong and established relationship between bedtime routines and sleep with sleep (Mindell et al., 2015; Mindell and Williamson, 2018). One subscale of the CSHQ, morning waking was significantly associated with bedtime routines indicating that better routines could result in easier morning waking behaviors for young children. Lack of general associations between sleep and bedtime routines could be attributed to the specific instruments used for this study.

### Achieving better bedtime routines; reflection on parents' experiences

Retrieving proper knowledge, through formal guidelines or family members' experiences, about bedtime routines is agreed to be essential. This could be seen as an expected result based on the sample characteristics: modern, first-time parents, mostly of upper education, becoming parents later than previous generations, usually as a conscious well-planned choice. Previous research on cross-cultural differences of families (Mindell et al., 2010) or immigrant populations (Hager et al., 2016; Lindsey, 2018) and low socioeconomic status households (Hale et al., 2011) show less interest or access in acquiring information and applying a well-structured routine. House environment and general resources is also a factor dependent on class characteristics (NICHD SECCYD-Wisconsin, 2014), which in the Greek sample is recognized but the effects seem to be regulated (noise, cold, house arrangement etc).

Based on the results of the qualitative study, the most important skills to facilitate a good routine are patience, perseverance, time management, regulation, and fatigue control. Mothers are more burdened with such responsibilities than fathers, putting them at greater risk of fatigue. That can be balanced through an increased belief in their capabilities to manage or can be worsened by beliefs of catastrophic consequences for the child's functioning and development. The latter are also referred to in short-term and long-term intentions and goals. Also, since our participants were mostly female, it is noted that mothers' mood and emotions are deeply affected by the outcome of the putting-to-bed procedure, as it is supported by previous research as well (Mindell et al., 2009).

A crucial aspect in the research of bedtime routines is the role they play in strengthening the parent-child relationship (Sadeh et al., 2010). An interesting finding is that a well-paced routine leading to positive experiences work as a mean of bond creation and as a reinforcement to sustain the routine. Although having a structured routine is acknowledged more as a habit, most agree that it is a flexible process following the developmental needs of the growing child and becoming less of parental responsibility and more of child self-regulated habit.

Social influence, mostly by close friends and family, can play a part in adopting practices both as a practical help and a source of recognition on the parental role, but are not beyond criticism. Such patterns are common in Greek cultural content, because of the close relationships with the community and family bonds. In this particular cultural content, it is also expected that traditional gender roles are maintained. Stereotypes and cognitions about the “role of the mother” lead to more intense occupation of women on child raising, including bedtime routines, even though women themselves stress out the important help of fathers in the procedure (Ganong and Coleman, 1995; Rentzou, 2011; Okimoto and Heilman, 2012).

Ultimately, addressing the key barriers identified by this work while reinforcing facilitators can lead to bedtime routines that are more stable, consistent during the week and the weekend and cover as many areas of interest as possible.

### Strengths and limitations

The present study has a series of strengths including: (a) being the first study of its kind within a Greek cohort of first-time parents, (b) utilizing a stepped approach starting with a general exploration of prevalence of routines before moving to more in-depth analyses with the deployment of qualitative interviews, (c) the use of a standardized qualitative framework to uncover key issues in achieving optimal and suboptimal routines and (d) the ability to harness current work and insights into future work and potential interventions due to the use of standardized frameworks. Despite its strengths, the current study has a series of limitations too including: (a) the composition and homogeneity of the sample which includes majority of participants from a higher educational background alongside the sample size for both studies can limit generalization of findings due to differences between our sample and overall characteristics of Greek families, (b) lack of paternal involvement in the study to allow for comparisons between different genders involved in child-rearing activities (however, in this study, responses related to the family bedtime routine not what each parent did with the child), (c) limitations associated with self-reported outcomes which could have resulted in desirability biases in reported data, (d) focus on first-time parents, even though it is important given the literature on learning something first, learn it right, focusing on first-time parents along limits the generalizability of findings, (e) the use of instruments not validated in Greek samples and/or not translated in Greek presented a further limitation to the study, (f) with regards to the CSHQ-abbreviated version, the age range for this instrument is slightly older children however, there is instances where different versions of the CSHQ have been used with younger children from 24-months old (Bonuck et al., 2017), (g) snowballing sampling for study 2 might have led to over-representation of parents who were more willing to talk about their bedtime routines and therefore risk of bias exists, (h) the overall small sample size of study 1 (*N* = 54) limited the depth of possible analyses we could perform and finally, (i) the lack of information regarding housing conditions (e.g., how many people live in each household, if child is sleeping in their own room etc.), information on family circumstances (e.g., single-parent families) and information on overall health including mental health of parents and children which could all have provided extremely useful insights to guide our analyses and strengthen our results.

### Implications for future work

Reflecting on this study's limitations, a more diverse sample should be included in future work to allow for better comparisons between gender, family types and socio-economic characteristics. Also, future work should consider deployment of more dynamic assessment of one's bedtime routines since they are a dynamic behavior including capturing the time bedtime routine activities start for a more complete data picture. Equally, validated and culturally appropriate data collections instruments, translated in the target language should be considered for more robust data. Finally, building on this work, consideration should be given on best ways to support families, especially first-time parents, as they start to establish and implement bedtime routines for their children. Interventions will need to reflect on the identified barriers while enhancing facilitators to sustain changes over time. Inclusive, user-led and user-involved designs should be followed to ensure that any future interventions and support offered to parents is working for them as intended.

## Data availability statement

The raw data supporting the conclusions of this article will be made available by the authors, without undue reservation.

## Ethics statement

The studies involving humans were approved by Ethics Committee of the Department of Psychology at the Aristotle University of Thessaloniki (Ref.: 059/06-05-2022). The studies were conducted in accordance with the local legislation and institutional requirements. The participants provided their written informed consent to participate in this study.

## Author contributions

MP: Data curation, Formal analysis, Investigation, Methodology, Writing—original draft, Writing—review & editing. MS: Data curation, Formal analysis, Investigation, Writing—original draft, Writing—review & editing. IK: Data curation, Formal analysis, Investigation, Writing—review & editing. NK: Data curation, Formal analysis, Investigation, Writing—review & editing. M-AM: Data curation, Formal analysis, Investigation, Writing—review & editing. FZ: Data curation, Formal analysis, Investigation, Writing—review & editing. GK: Conceptualization, Formal analysis, Methodology, Supervision, Writing—original draft, Writing—review & editing.
